# Non‐communicable diseases by age strata in people living with and without HIV in four African countries

**DOI:** 10.1002/jia2.25985

**Published:** 2022-09-29

**Authors:** David Chang, Allahna L. Esber, Nicole F. Dear, Michael Iroezindu, Emmanuel Bahemana, Hannah Kibuuka, John Owuoth, Jonah Maswai, Trevor A. Crowell, Christina S. Polyak, Joseph S. Cavanaugh, Julie A. Ake, Catherine Godfrey, the AFRICOS Study Group

**Affiliations:** ^1^ U.S. Military HIV Research Program, Walter Reed Army Institute of Research Silver Spring Maryland USA; ^2^ Henry M. Jackson Foundation for the Advancement of Military Medicine Inc. Bethesda Maryland USA; ^3^ HJF Medical Research International Abuja Nigeria; ^4^ HJF Medical Research International Mbeya Tanzania; ^5^ Makerere University Walter Reed Project Kampala Uganda; ^6^ U.S. Army Medical Research Directorate – Africa Kisumu Kenya; ^7^ HJF Medical Research International Kisumu Kenya; ^8^ HJF Medical Research International Kericho Kenya; ^9^ Department of State Office of the Global AIDS Coordinator Washington DC USA

**Keywords:** Africa, LMIC, HIV epidemiology, quality of life, cohort studies, HIV care continuum

## Abstract

**Introduction:**

Non‐communicable diseases (NCDs) are an important driver of morbidity among ageing people living with HIV (PLWH). We examined the composite role of age and HIV status on NCDs in people living with and without HIV.

**Methods:**

The African Cohort Study (AFRICOS) prospectively enrols participants aged ≥15 years with and without HIV at 12 sites in Kenya, Tanzania, Uganda and Nigeria. From 21 January 2013 to 1 September 2021, we assessed participants for renal insufficiency (estimated glomerular filtration rate <60 ml/minute/1.73 m^2^), elevated blood pressure (BP) (any systolic BP >139 mmHg or diastolic BP >89 mmHg), obesity (body mass index >30 kg/m^2^), diabetes mellitus (DM) (fasting glucose ≥126 mg/dl or antidiabetic medication) and dysglycemia (fasting glucose ≥99 mg/dl or non‐fasting ≥199 mg/dl). Multivariable logistic regression with generalized estimating equations was used to estimate odds ratios (ORs) and 95% confidence intervals (CIs) for factors associated with each NCD. The main exposure of interest was a composite of HIV status and age dichotomized around 50 years. All models were adjusted for study site and sex. The renal insufficiency model was additionally adjusted for elevated BP and dysglycemia.

**Results and discussion:**

Of 3761 participants with age data, 557 (14.8%) were age ≥50, 2188 (58.2%) were females and 3099 (82.4%) were PLWH. At enrolment, the prevalence of elevated BP, dysglycemia, renal insufficiency and obesity were *n* = 128 (26.9%), *n* = 75 (15.8%), *n* = 8 (1.7%) and *n* = 40 (8.4%), respectively, for PLWH ≥50. Compared to people without HIV age <50, PLWH age ≥50 had increased adjusted odds of having DM (OR: 2.78, 95% CI: 1.49–5.16), dysglycemia (OR: 1.98, 95% CI: 1.51–2.61) and renal insufficiency (OR: 6.20, 95% CI: 2.31–16.66). There were significant differences by study site, specifically, participants from Nigeria had the highest odds of elevated BP, dysglycemia and renal insufficiency as compared to Uganda.

**Conclusions:**

There was a high burden of NCDs in this African cohort with differences by geographic region. In order to promote healthy ageing with HIV, screening and treatment for common NCDs should be incorporated into routine HIV care with attention paid to geographic heterogeneity to better allocate resources.

## 1 INTRODUCTION

People living with HIV (PLWH) are experiencing increased life expectancy in both high‐ and low‐income countries with some life expectancy nearing that of people living without HIV (PLWoH) [1, 2, 3, 4, 5]. As the access to antiretroviral therapy (ART) is scaled up and progress is made in reaching UNAIDS 95‐95‐95 targets, the median age of PLWH is expected to increase. Recent data demonstrate that one‐third of PLWH in the U.S. President's Emergency Plan for AIDS Relief (PEPFAR) programmes are age >50 [6]. As the PLWH population ages, it is important to understand the effect of HIV and age to promote healthy ageing.

Non‐communicable diseases (NCDs), such as diabetes and cardiovascular disease, have been associated with HIV and are important drivers of morbidity and mortality [7, 8, 9]. Studies show that PLWH in sub‐Saharan Africa have a high prevalence of NCDs, such as hypertension [10]. PLWH appear to have a higher prevalence of NCDs, such as hyperglycaemia and diabetes, as compared to PLWoH [11, 12]. Previous work from the African Cohort Study (AFRICOS) showed that PLWH on ART have an increased risk of NCDs compared to PLWH not on ART [13]. We investigated the prevalence and factors associated with NCDs in AFRICOS, focusing on age and HIV status.

## 2 METHODS

### 2.1 Study setting and population

AFRICOS is an ongoing prospective cohort enrolling at 12 clinics across five programmes supported by PEPFAR: Kayunga, Uganda; South Rift Valley, Kenya; Kisumu West, Kenya; Mbeya, Tanzania; and Lagos and Abuja, Nigeria [13]. PLWH were recruited from randomized lists of current PEPFAR clinic patients and those with new HIV diagnoses. Enrolees were encouraged to bring partners in for testing and recruitment. PLWoH were also recruited from community members accessing HIV testing, a small subset of participants was recruited from prior research studies. Participants were included if they were age ≥15 years, intended to be a long‐term area resident, willing to provide contact information, consented to data/specimen collection and storage for future use, and understood English or the local language. Individuals were excluded if they were pregnant at enrolment.

The study was approved by institutional review boards of the Walter Reed Army Institute of Research and all collaborating institutions. All participants provided written informed consent.

### 2.2 Procedures

At enrollment, all participants were administered a medical history, physical exam, demographic questionnaire and underwent phlebotomy. PLWH underwent confirmatory HIV rapid diagnostic testing, CD4 T‐lymphocyte count and HIV Viral Load (VL) [14]. Study visits occurred every 6 months and participants provided medical history, completed a physical examination and underwent laboratory assessments. Study clinicians performed medical record reviews and extracted ART start date and regimen at every visit. HIV rapid tests were performed at each visit for PLWoH and CD4 counts and VL were performed at every visit for PLWH. All participants had an assessment of serum creatinine and blood glucose performed annually. Study‐specific laboratory assessments included tests that were not part of routine care at study sites; test results were shared with care providers. All assessments were performed in laboratories that were accredited by the College of American Pathologists or had successfully completed external quality assurance.

### 2.3 Data collection and definitions

Demographic variables, including sex, age, education level, HIV status and clinical care site, are reported for the enrolment visit. For PLWH, ART use and VL stratum were combined into the following categories: not on ART, on ART and VL <1000 copies/ml, and on ART and VL ≥1000 copies/ml. CD4 nadir was categorized as <200, 200–349, 350–499 and ≥500 cells/mm^3^. All data were recorded on paper case report forms and double entered into the ClinPlus platform (DZS Software Solutions, Bound Brock, NJ).

Elevated blood pressure (BP) was defined as systolic blood pressure of >139 mmHg, diastolic blood pressure >89 mmHg or receipt of antihypertensive medications. Abnormal BPs were repeated for confirmation. Dysglycemia was defined as fasting glucose ≥99 mg/dl, non‐fasting glucose ≥199 mg/dl or receipt of hypoglycaemic medications. Diabetes mellitus (DM) was defined as fasting glucose ≥126 mg/dl or receipt of hypoglycaemic medications. Renal insufficiency was defined as the estimated glomerular filtration rate <60 ml/minute/1.73 m^2^ calculated using the Modification of Diet in Renal Disease equation [15]. Obesity was defined as a body mass index (BMI) of >30 kg/m^2^.

### 2.4 Statistical analyses

The main exposure of interest was a composite of HIV status and age dichotomized around 50 years. Comparisons of demographic characteristics and other parameters across groups of interest were made using Pearson chi‐squared test for categorical variables and the Kruskal–Wallis test for continuous variables. Longitudinal analyses involved multivariable logistic regression with generalized estimating equations, clustered by a participant to account for repeated measures, to estimate odds ratios (ORs) and 95% confidence intervals (CIs) for factors associated with each NCD. NCDs were assessed at every follow‐up visit and the model was time updated. Once diagnosed with an NCD, a participant would not have additional events of that disease. DM and dysglycemia models were adjusted for potential confounders, including study site and sex. Renal insufficiency models were adjusted for elevated BP and dysglycemia given the known risk factors for disease [16, 17]. Analyses were performed in SAS 9.3 (SAS, Cary, NC) and Stata 16.0 (StataCorp, College Station, TX).

## 3 RESULTS AND DISCUSSION

### 3.1 Demographics and clinical characteristics

Between 21 January 2013 and 1 September 2021, 3762 participants were enrolled in AFRICOS and 3761 with age data were included in these analyses (Table 1). Data from the enrolment visit are presented in Table 1. Most of the cohort were PLWH, 3099 (82.4%). There were fewer participants aged ≥50, with the least comprised of PLWoH age ≥50 (*n* = 80, 2.1%). Among PLWH age <50, 1572 (60.0%) had virologic suppression less than 1000 copies/ml. In comparison, a greater proportion of PLWH age ≥50 had virologic suppression less than 1000 copies/ml (*n* = 351, 73.6%). Thirty‐six percent (*n* = 1135) of participants had a CD4 nadir below 200 cells/mm^3^. Thirty‐five percent (*n* = 1099) had an enrolment CD4 above 500 cells/mm^3^. Most PLWH were on a non‐nucleoside reverse transcriptase inhibitor or an integrase strand transfer inhibitor (INSTI); only 5.5% were on a protease inhibitor based regimen.

**Table 1:** Demographic and clinical characteristics of study participants stratified by age and HIV status at enrolment

	<50, PLWoH	<50, PLWH	≥50, PLWoH	≥50, PLWH	Total	
	*n* = 582	*n* = 2622	*n* = 80	*n* = 477	*N* = 3761	*p*‐value
Study site						<0.001
Kayunga, Uganda	95 (16.3%)	478 (18.2%)	18 (22.5%)	72 (15.1%)	663 (17.6%)	
South Rift Valley, Kenya	176 (30.2%)	881 (33.6%)	32 (40.0%)	168 (35.2%)	1257 (33.4%)	
Kisumu West, Kenya	123 (21.1%)	448 (17.1%)	17 (21.3%)	103 (21.6%)	691 (18.4%)	
Mbeya, Tanzania	91 (15.6%)	488 (18.6%)	6 (7.5%)	109 (22.9%)	694 (18.5%)	
Abuja and Lagos Nigeria	97 (16.7%)	327 (12.5%)	7 (8.8%)	25 (5.2%)	456 (12.1%)	
Sex						<0.001
Male	249 (42.8%)	1007 (38.4%)	41 (51.2%)	276 (57.9%)	1573 (41.8%)	
Female	333 (57.2%)	1615 (61.6%)	39 (48.8%)	201 (42.1%)	2188 (58.2%)	
Age (years), median (IQR)	32 (25.3–39)	35.4 (28.4–41.5)	54.8 (52.45–58.75)	54.8 (52.2–58.7)	37 (29.2–45.1)	<0.001
Education						<0.001
Primary or less	266 (45.7%)	1451 (55.3%)	47 (58.8%)	306 (64.2%)	2070 (55.0%)	
Secondary or above	315 (54.1%)	1168 (44.5%)	33 (41.3%)	171 (35.8%)	1687 (44.9%)	
Missing	1 (0.2%)	3 (0.1%)	0 (0.0%)	0 (0.0%)	4 (0.1%)	
BMI 30+						<0.001
No	500 (85.9%)	2456 (93.7%)	66 (82.5%)	436 (91.4%)	3458 (91.9%)	
Yes	81 (13.9%)	161 (6.1%)	14 (17.5%)	40 (8.4%)	296 (7.9%)	
Missing	1 (0.2%)	5 (0.2%)	0 (0.0%)	1 (0.2%)	7 (0.2%)	
Diabetes						<0.001
No	293 (50.3%)	2546 (97.1%)	38 (47.5%)	457 (95.8%)	3334 (88.6%)	
Yes	8 (1.4%)	31 (1.2%)	2 (2.5%)	18 (3.8%)	59 (1.6%)	
Missing	281 (48.3%)	45 (1.7%)	40 (50.0%)	2 (0.4%)	368 (9.8%)	
Dysglycemia						<0.001
No	275 (47.3%)	2360 (90.0%)	33 (41.3%)	400 (83.9%)	3068 (81.6%)	
Yes	26 (4.5%)	217 (8.3%)	7 (8.8%)	75 (15.7%)	325 (8.6%)	
Missing	281 (48.3%)	45 (1.7%)	40 (50.0%)	2 (0.4%)	368 (9.8%)	
Renal insufficiency						0.35
No	296 (50.9%)	2562 (97.7%)	39 (48.8%)	469 (98.3%)	3366 (89.5%)	
Yes	1 (0.2%)	31 (1.2%)	0 (0.0%)	8 (1.7%)	40 (1.1%)	
Missing	285 (49.0%)	29 (1.1%)	41 (51.2%)	0 (0.0%)	355 (9.4%)	
Elevated BP						<0.001
No	503 (86.4%)	2371 (90.4%)	49 (61.3%)	348 (73.0%)	3271 (87.0%)	
Yes	79 (13.6%)	247 (9.4%)	31 (38.8%)	128 (26.8%)	485 (12.9%)	
Missing	0 (0.0%)	4 (0.2%)	0 (0.0%)	1 (0.2%)	5 (0.1%)	
ART type						<0.001
EFV		959 (36.6%)		201 (42.1%)	1160 (37.4%)	
NVP		521 (19.9%)		169 (35.4%)	690 (22.3%)	
DTG		159 (6.1%)		0 (0.0%)	159 (5.1%)	
PI		148 (5.6%)		22 (4.6%)	170 (5.5%)	
ART naïve		827 (31.5%)		82 (17.2%)	909 (29.3%)	
Other		8 (0.3%)		3 (0.6%)	11 (0.4%)	
CD4 count nadir (cells/mm^3^)						<0.001
<200		906 (34.6%)		229 (48.0%)	1135 (36.6%)	
200–349		538 (20.5%)		111 (23.3%)	649 (20.9%)	
350–499		259 (9.9%)		37 (7.8%)	296 (9.6%)	
500+		268 (10.2%)		28 (5.9%)	296 (9.6%)	
Missing		651 (24.8%)		72 (15.1%)	723 (23.3%)	
CD4 count (cells/mm^3^)						0.23
<200		479 (18.3%)		96 (20.1%)	575 (18.6%)	
200–349		600 (22.9%)		123 (25.8%)	723 (23.3%)	
350–499		563 (21.5%)		101 (21.2%)	664 (21.4%)	
500+		947 (36.1%)		152 (31.9%)	1099 (35.5%)	
Missing		33 (1.3%)		5 (1.0%)	38 (1.2%)	
Duration on ART						<0.001
<6 months		379 (14.5%)		52 (10.9%)	431 (13.9%)	
6 months–5 years		797 (30.4%)		166 (34.8%)	963 (31.1%)	
5+ years		602 (23.0%)		173 (36.3%)	775 (25.0%)	
ART naïve		827 (31.5%)		82 (17.2%)	909 (29.3%)	
Missing		17 (0.6%)		4 (0.8%)	21 (0.7%)	
Viral suppression <1000 copies/ml						<0.001
Not suppressed		995 (37.9%)		122 (25.6%)	1117 (36.0%)	
Suppressed		1572 (60.0%)		351 (73.6%)	1923 (62.1%)	
Missing		55 (2.1%)		4 (0.8%)	59 (1.9%)	

Note: Participant characteristics at enrolment, by age and HIV status. Significant differences between the four age/HIV status groups were assessed using Pearson chi‐squared tests for categorical variables and Kruskal–Wallis for continuous variables to identify whether the proportion with a particular characteristic is different in one or more groups as compared to the others.

Abbreviations: ART, antiretroviral therapy; DTG, dolutegravir; EFV, efavirenz; NVP, nevirapine; PI, protease inhibitor; PLWH, people living with HIV; PLWoH, people living without HIV.

### 3.2 Prevalence of NCDs

**Figure 1:**
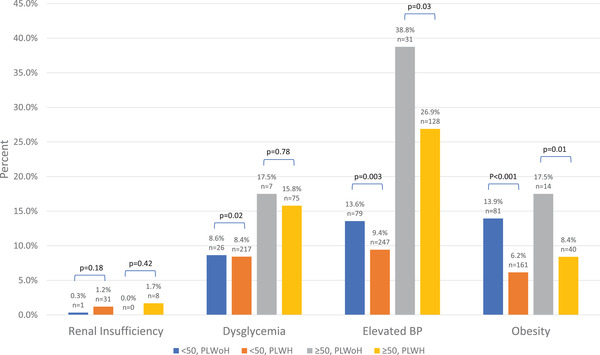
Prevalence of non‐communicable diseases at enrolment. The prevalence of NCDs by age and HIV status at enrolment visit. Abbreviations: PLWH, people living with HIV; PLWoH, people living without HIV; BP, blood pressure.

There was a higher prevalence of NCDs in the ≥50 age group for both PLWH and PLWoH at enrolment (Figure 1). In PLWH age ≥50, the most common NCD was elevated BP (26.9%), followed by dysglycemia (15.8%), obesity (8.4%) and renal insufficiency (1.7%). PLWoH had a statistically significant increased prevalence of elevated BP and obesity when compared to PLWH in the same age group. There were no significant differences seen between PLWoH and PLWH age ≥50 for renal insufficiency and dysglycemia.

### 3.3 Factors associated with NCDs

Participants were followed for a median (IQR) of 4.66 (1.74–6.08) years to evaluate factors associated with NCDs. In the multivariable analysis for factors associated with dysglycemia and diabetes (Table 2), there were significant increases in odds of having both diseases in PLWH and PLWoH age ≥50 when compared to PLWoH age <50. There were no significant differences when comparing PLWH age <50 to PLWoH age <50. Study site differences were seen for dysglycemia and diabetes. Participants in Nigeria had 4.66 (95% CI: 3.52–6.17) and 2.12 (95% CI: 1.13–3.99) odds of having dysglycemia and diabetes, respectively, compared to participants in Uganda. Participants in Kenya and Tanzania also had significantly higher odds of having dysglycemia as compared to participants in Uganda. BMI >30 was significantly associated with dysglycemia and diabetes.

**Table 2:** Adjusted odds of non‐communicable diseases

	Dysglycemia				Diabetes				Renal insufficiency			
	no. ppts (*n* = 3623)	no. obs (*n* = 15,433)	aOR	95% CI	no. ppts (*n* = 3623)	no. obs (*n* = 15,430)	aOR	95% CI	no. ppts (*n* = 3620)	no. obs (*n* = 15,406)	aOR	95% CI
Age, HIV status												
<50, PLWoH	66/470	83/1050	–		17/470	20/1049	–		7/469	4/1044	–	
<50, PLWH	640/2607	988/10,878	1.19	0.93–1.54	124/2607	176/10,876	0.88	0.48–1.60	169/2606	154/10,870	3.56	1.35–9.40
≥50, PLWoH	18/70	49/255	2.61	1.70–4.02	9/70	33/255	6.75	3.47–13.14	7/69	7/243	7.64	2.36–24.67
≥50, PLWH	177/476	467/3250	1.98	1.51–2.61	60/476	167/3250	2.78	1.49–5.16	64/476	76/3249	6.20	2.31–16.66
Study site												
Uganda	92/641	150/3052	–		24/641	54/3052	–		25/640	27/3043	–	
SRV, Kenya	369/1210	648/5162	2.40	1.81–3.17	78/1210	171/5161	1.69	0.92–3.12	87/1209	66/5146	1.25	0.66–2.36
Kisumu, Kenya	96/673	117/2869	0.81	0.58–1.13	26/673	29/2869	0.56	0.29–1.09	29/673	32/2870	1.20	0.57–2.54
Tanzania	125/657	215/2382	1.70	1.23–2.35	37/657	74/2381	1.55	0.79–3.00	31/658	36/2383	1.42	0.69–2.89
Nigeria	219/442	457/1968	4.66	3.52–6.17	45/442	68/1967	2.12	1.13–3.99	75/440	80/1964	4.00	2.12–7.53
Sex												
Male	423/1510	776/6389	–		103/1510	192/6389	–		81/1509	87/6378	–	
Female	478/2113	811/9044	0.71	0.62–0.82	107/2113	204/9041	0.75	0.51–1.11	166/2111	154/9028	1.42	0.99–2.04
BMI												
BMI <30	809/3346	1297/13,845	–		174/3346	302/13,843	–					
BMI >30	92/277	290/1588	1.74	1.47–2.06	36/277	94/1587	2.40	1.56–3.68				
Elevated BP												
No									180/3161	167/13,192	–	
Yes									67/459	74/2214	1.91	1.32–2.76
Dysglycemia												
No									209/3275	192/13,838	–	
Yes									38/345	49/1568	1.48	1.00–2.19

Note: Dysglycemia and DM models were adjusted for age/HIV status, study site, sex and BMI. The renal insufficiency model was adjusted for age/HIV status, study site, sex, dysglycemia and elevated BP.

Abbreviations: BMI, body mass index; PLWH, people living with HIV; PLWoH, people living without HIV; no. ppt, number of participants; no obs, number of observations during follow up; SRV, South Rift Valley.

The multivariable analysis for renal insufficiency (Table 2) demonstrated increased odds for PLWH age <50, PLWoH and PLWH age ≥50 of having renal insufficiency when compared to PLWoH age <50. The greatest odds of disease were seen in PLWoH age ≥50 with wide CIs overlapping with the PLWH age ≥50 CI's. Study site differences were again seen with participants in Nigeria having 4.0 (95% CI: 2.12–7.53) odds of having renal insufficiency compared to participants in Uganda. Participants with elevated BP and dysglycemia had increased odds of having renal insufficiency, 1.91 (95% CI: 1.32–2.76) and 1.48 (95% CI: 1.00–2.19), respectively.

## 4 DISCUSSION

NCDs are a leading cause of morbidity and mortality in low‐ and middle‐income countries [7, 18]. In this observational cohort in four sub‐Saharan African countries, there was a high burden of NCDs. Over a quarter of PLWH age ≥50 had elevated BP and over 15% had dysglycemia. Factors associated with NCDs were consistent with those known to be a risk factor for NCDs, such as obesity increasing the risk for diabetes or diabetes increasing the risk for renal insufficiency [16, 17].

Geographic heterogeneity was seen with NCDs in AFRICOS. Cohort participants in Nigeria had the highest odds of having dysglycemia and renal insufficiency. NCDs account for up to 29% of all deaths in Nigeria [19]. Dietary differences could account for the differences seen in renal insufficiency as Nigerians appear to have higher salt intake than recommended [20]. The study sites in Nigeria were only in urban centres, which may explain dietary and physical activity differences potentially accounting for differences seen in NCDs [21]. Further analyses will be needed to understand the aetiology for the geographic heterogeneity seen and to better guide interventions.

Treatment of HIV with ART has been implicated in the development of certain NCDs by side effects or toxicity. Long‐term use of tenofovir disoproxil fumarate (TDF) can result in nephrotoxicity. Dolutegravir raises serum creatinine without changing renal function because it inhibits proximal renal tubular secretion of creatinine by organic cation transporters [22]. In addition, the increased odds of renal insufficiency at a younger age in PLWH may be due to “accelerated ageing” where conditions seen in older persons appear in PLWH at a younger age [8, 23]. In this cohort, there was an association seen with renal insufficiency in the age <50 group with PLWH having increased odds of having disease compared to PLWoH. While this analysis did not focus on aetiology, the risk of renal disease in PLWH can be modified by changing ART regimens. Most participants in this cohort are on TDF and considerations can be made to switch to newer, less nephrotoxic, tenofovir formulations in areas of high renal insufficiency prevalence.

INSTIs, particularly second‐generation INSTIs, have been implicated in substantial weight gain [24, 25, 26]. While over 70% of PLWH age ≥50 were on an INSTI at the most recent visit, DTG was not programmatically rolled out by PEPFAR until late 2018 [27]. The maximum exposure time of DTG at the time of this analysis would have been 3 years. Even though the prevalence of obesity in PLWH age ≥50 was significantly lower compared to age‐matched counterparts without HIV, the prevalence of obesity should be monitored as the duration of DTG increases. If the evidence for DTG association with weight gain becomes stronger, programmatic considerations can be considered weighing the benefits of DTG against other options.

This study has multiple strengths, including a diverse population with extensive data collected since 2013. Limitations are that this is an observational cohort with a low number of participants aged≥50, particularly PLWoH in that age group that limit statistical power. Testing for diabetes, dysglycemia and renal insufficiency was not added to study procedures for PLWoH until 2017 and, therefore, these data are not available for these participants. However, data were included for subsequent visits by these participants after amendment implementation. We attempted to adjust for confounders, including study site, age, HIV status, sex and other diseases, known to increase the risk of NCDs; however, we did not adjust for ART exposure differences given the inclusion of PLWoH. We were unable to adjust for other confounders, such as physical activity and nutrition, as these were not collected as part of the study.

## 5 CONCLUSIONS

This study provides NCD prevalence and characteristics in an ageing sub‐Saharan African PLWH population. There was a large burden of NCDs in this cohort that varied by geographic region. If left unmitigated, NCDs can lead to downstream effects, such as neurologic and cardiovascular disease, causing significant morbidity and mortality. Treating individuals with multiple comorbidities is complex and models of care will need to be developed to appropriately manage these individuals. The geographic component is important because in areas with high burdens of NCDs it will be important to appropriately allocate resources to promote healthy ageing with HIV.

## COMPETING INTERESTS

The authors have no competing interests to disclose.

## AUTHORS’ CONTRIBUTIONS

CCG, JAA, JSC, ALE and DC conceived of the presented research idea. EB, MI, HK, JO, JM and VS carried out the data collection, laboratory activities and reviewed the collected data for quality and reliability. ALE designed the model and analysed the data. ND verified underlying data. DC, ALE, JAA, TAC, NFD, CSP, JSC and CCG contributed to the interpretation of the results. ALE and DC took the lead in writing the manuscript. CCG, CSP and JAA were in charge of overall direction and planning. All authors provided critical feedback and helped shape the research, analysis and manuscript. All authors approve of the final submitted manuscript.

## FUNDING

This work was supported by the President's Emergency Plan for AIDS Relief via a cooperative agreement between the Henry M. Jackson Foundation for the Advancement of Military Medicine, Inc. and the U.S. Department of Defense.

## DISCLAIMER

The views expressed are those of the authors and should not be construed to represent the positions of the US Army, the Department of Defense or the Department of State. The investigators have adhered to the policies for the protection of human subjects as prescribed in Army Regulation 70‐25.

## Data Availability

The datasets generated and/or analysed during the current study are not publicly available due to privacy protections but are available from the corresponding author on reasonable request. The Henry M. Jackson Foundation for the Advancement of Military Medicine (HJF) and the Water Reed Army Institute of Research (WRAIR) are committed to safeguarding the privacy of research participants. The distribution of data will require compliance with all applicable regulatory and ethical processes, including the establishment and approval of an appropriate data‐sharing agreement. To request a minimal dataset, please contact the data coordinating and analysis center (DCAC) at PubRequest@hivresearch.org and indicate the RV329 study along with the name of the manuscript.
